# Use of quality-of-care and patient-safety policies in the WHO European region: an ecological cross-sectional exploratory analysis of policy adoption and health outcomes

**DOI:** 10.3389/fpubh.2026.1904393

**Published:** 2026-10-12

**Authors:** Liesbeth Borgermans, Ana Marques Domingues, Anna Crispo, Válter R. Fonseca, Lenio Capsaskis, Jennifer Hall, João Breda

**Affiliations:** Epidemiology and Biostatistics Unit, Istituto Nazionale Tumori IRCCS “Fondazione G. Pascale”, Naples, Italy

**Keywords:** quality of care, patient safety, policies, health outcomes, WHO European Region

## Abstract

**Objective:**

To assess the implementation of quality-of-care and patient-safety policies across the WHO European Region and their association with population health outcomes.

**Methods:**

This study used an exploratory, ecological cross-sectional design. We constructed a summary count (0–9) of nine national quality-of-care and patient-safety policies to capture the breadth of policy adoption, and used exploratory multivariable linear regression to examine cross-sectional associations with selected outcomes, adjusting for health-workforce density and public spending on health.

**Results:**

Many countries lacked national action plans for quality and patient safety, with only around one-third implementing both. Implementation of a national quality-of-care plan was associated with higher healthy life expectancy (1.68 additional years; *p*_unadjusted_ = 0.033, *p* = 0.144), a lower probability of premature death from cardiovascular disease, cancer, diabetes and chronic respiratory diseases (−3.33 percentage points; *p*_unadjusted_ = 0.049, *p* = 0.144), and reduced under-five mortality (−4.06 deaths per 1,000 live births; *p*_unadjusted_ = 0.023, *p* = 0.144). Implementing a greater number of national policies was associated with small increases in healthy life expectancy (0.29 years per additional policy; *p*_unadjusted_ = 0.043, *p* = 0.167) and lower under-five mortality (−0.72 deaths per 1,000 live births per additional policy; *p*_unadjusted_ = 0.018, *p* = 0.162), while associations with other outcomes were weaker and often non-significant.

**Conclusion:**

Given the ecological cross-sectional design, limited adjustment and multiple testing, these findings should be treated as exploratory and non-causal and used as a basis for more detailed longitudinal research.

## Introduction

Universal access to high-quality healthcare without financial hardship is a key objective of the World Health Organization (WHO) Regional Office for Europe (1). This priority is embedded in the second European Program of Work 2026–2030 (EPW2) (2) and in the 2030 Agenda for Sustainable Development (3), which emphasize universal access to essential health services without financial hardship. This ensures that every individual and community is able to access the health services they need, whether they are promotional, preventive, curative, rehabilitative or palliative, without suffering financial distress. As service use and coverage have expanded across the region, the importance of quality of care in achieving improved health outcomes and broader societal progress has become increasingly recognized. High-quality care involves providing services that are safe, effective, timely, patient-centered, efficient and equitable, increasing the likelihood of better health outcomes, patient satisfaction and fair access to services (4, 5).

However, persistent shortcomings in quality of care and patient safety continue to affect the most vulnerable groups in particular (6). In low- and middle-income countries, poor-quality medical care is responsible for up to 58% of preventable deaths, surpassing the health impacts of insufficient access (6). In high-income countries, data indicate that 1 in 10 patients experiences an adverse and preventable event during hospitalization (7). Furthermore, the direct costs of treating patients who have been harmed by their care can account for up to 13% of healthcare expenditure (7). Substandard care also has wider social and economic consequences, including catastrophic health expenditure and higher costs for expanding coverage (7).

Leadership and governance are critical for ensuring quality of care and patient safety because they set the vision, create the culture, establish accountability and coordinate the resources necessary for effective healthcare delivery (8). Effective leadership is fundamental to building resilient, high-quality systems (6) that consistently improve or maintain health outcomes, earn the trust of the population and adapt to evolving needs (9–11).

Effective leaders invest in workforce capacity, digital infrastructure, and innovation, while embedding patient and community engagement in decision-making processes (12). By strengthening data and measurement systems, promoting transparency, and fostering multi-sectoral partnerships, leaders translate strategic priorities into measurable improvements in quality of care, patient safety, and population health outcomes.

The WHO Regional Office for Europe recently conducted a region-wide assessment of quality of care and patient safety across its 53 Member States, revealing substantial variation in performance (4). Building on this work, the present article derives a pragmatic summary measure of national adoption across nine quality-of-care and patient-safety policy instruments and explores how this measure relates to selected population-health outcomes using cross-sectional regression models. The analysis is not designed to provide causal estimates or a validated index of policy performance; rather, it is intended to generate hypotheses about potential associations and to identify patterns in a population-level setting that can inform future, more detailed studies.

## Methods

We conducted an exploratory cross-sectional ecological analysis across the WHO European Region, with individual Member States as the unit of analysis. The analysis combined country-level data on the adoption and implementation of national quality-of-care and patient-safety policies, collected through a standardized WHO survey in 2024, with routinely available country-level population health indicators obtained from international databases. For each outcome, the most recent data available at the time of analysis were used. The study was designed to characterize variation in the breadth of national policy adoption across the Region and to explore whether countries with more comprehensive quality-of-care and patient-safety policy frameworks also tended to report more favorable population health outcomes.

The analysis was exploratory and hypothesis-generating rather than confirmatory. Exposures, covariates and outcomes were measured and analyzed at the aggregate country level; consequently, observed associations describe relationships between national policy environments and population-level health indicators and cannot be interpreted as associations at the individual, patient, provider or facility level. In addition, because policy data and outcome indicators were not necessarily measured in the same calendar year, and because the cross-sectional design does not establish the temporal sequence between policy adoption and observed outcomes, the results do not support causal inference regarding the effects of policy implementation. Ecological analyses are valuable for identifying population-level and health-system patterns, generating hypotheses, and informing further investigation; however, they remain susceptible to ecological fallacy, residual and unmeasured confounding, reverse causation, differences in national data availability and measurement practices, and limited ability to assess policy implementation intensity, quality, duration or enforcement (13, 14). The findings should therefore be interpreted as descriptive associations that can inform more detailed longitudinal, quasi-experimental and individual-level research.

The qualitative indicators collected across the 53 Member States of the WHO European Region in 2024 included national quality of care strategies/action plans, national patient safety plans, hospital accreditation systems, national plans on antimicrobial resistance (AMR), mechanisms for patient/public representation in governance, health misinformation prevention plans, national electronic health record systems, the use of quality and safety in telehealth guidelines, and a national list of approved priority or essential medical devices (4). Together, these policies form a comprehensive package of governance and system-strengthening measures, and the extent to which they have been adopted signals a greater level of commitment and capacity across the system.

Data were collected using a standardized survey distributed to and completed by the Quality of care and Patient Safety country focal point in each Member State. In cases where no focal point had previously been designated, an expert in the respective field was appointed by the relevant national health authorities to complete the survey. In addition to the survey data, the indicator “National antimicrobial resistance plan” was included, with data retrieved from the report Antimicrobial resistance surveillance in Europe, 2023 (15). The analysis included 51 countries: 44 countries with survey responses and 7 countries for which data were available only for indicator “National antimicrobial resistance plan.” Survey responses were received from 44 Member States: Albania, Armenia, Austria, Azerbaijan, Belarus, Belgium, Bosnia and Herzegovina, Bulgaria, Croatia, Cyprus, Czechia, Denmark, Estonia, Finland, France, Georgia, Germany, Greece, Iceland, Ireland, Israel, Italy, Kazakhstan, Kyrgyzstan, Latvia, Luxembourg, Malta, Montenegro, Netherlands, North Macedonia, Norway, Poland, Portugal, Republic of Moldova, Romania, Serbia, Slovakia, Slovenia, Spain, Sweden, Switzerland, Türkiye, Ukraine and United Kingdom.

For each indicator, respondents were asked to select one of four response categories: “Yes,” “Yes, in progress”, “No” or “No data available”. They were also asked to provide the relevant source(s) and supporting documentation, recognized and validated by the relevant national authorities, to substantiate their responses. The survey responses and documentations were subsequently reviewed and validated by a working group from the WHO Athens Quality of Care and Patient Safety Office. The validation process included reviewing the submitted supporting documentation and assessing the consistency of the survey responses with the available evidence. In addition, consultations with country focal points were used to clarify or verify information where necessary. The analysis included descriptive statistics summarizing policy implementation across countries and subregions. Country groupings for analysis were based on established WHO European subregions: EU15 countries (EU members before May 2004), EU13 countries (EU members after May 2004), CIS countries (Commonwealth of Independent States), South-Eastern European Health Network (SEEHN) countries and a “Rest of the Region” group. These groupings allow for the comparison of policy development and implementation according to health system maturity and socio-economic context.

We used adjusted multivariable linear regression to examine associations between the number of implemented quality-of-care and patient-safety policies and the following population health outcomes: healthy life expectancy at birth (years) (16); probability of dying from cardiovascular diseases, cancer, diabetes or chronic respiratory diseases (years) (17); standardized preventable mortality rate (years) (18); standardized treatable mortality rate (years) (18); average length of stay in all hospitals (days, years) (19); surgical wound infection rate for all operations (percentage per year) (20); influenza vaccination coverage (average and in the poorest quintile, percentage over selected years) (21); under-five mortality rate (per 1,000 live births) (22); and maternal mortality rate (per 100,000 live births) (23). Given the cross-sectional design and the limited sample of 53 countries, models were specified parsimoniously and adjusted only for a small set of structural and resource-related covariates—medical doctors per 10,000 population, nursing personnel per 10,000 population and public spending on health as a share of total public expenditure—which have been shown to influence outcomes such as life expectancy and infant mortality (24). We did not include additional socio-economic or governance indicators in order to preserve statistical power, and therefore anticipate residual confounding by broader health-system development; the estimated coefficients should be interpreted as descriptive associations rather than causal effects.

Most of the dependent variables analyzed in this study (healthy life expectancy, mortality rates, under-five and maternal mortality, and average length of stay) are continuous or approximately continuous at the country level, which supports the use of ordinary least squares (OLS) regression as a reasonable first approximation. The probability of dying between ages 30 and 70 from major noncommunicable diseases is modeled as a continuous percentage. To assess the robustness of our findings to outcome distributions and modeling assumptions, we conducted additional analyses using log-transformed outcomes for skewed variables and estimated models with robust standard errors. These checks are summarized in Supplementary Table S1. Given the sample size, we did not estimate more complex non-linear or multilevel models, which limits our ability to fully accommodate the distributional properties of all outcomes.

Observations with missing values were excluded from the analyses listed above.

We created a summary policy count to capture the extent to which quality-of-care and patient-safety measures were adopted nationally across the nine aforementioned domains. For the purposes of the analysis, each policy was dichotomized as “implemented” or “not implemented.” Responses coded as “Yes” were classified as implemented, whereas responses coded as “Yes, in progress”, “No”, or “No data available” were classified as not implemented. This conservative classification was adopted to ensure that only policies for which clear evidence of implementation was available were considered implemented. Policies reported as being in progress were not assumed to be fully implemented, while responses indicating that no data were available were classified as not implemented because the available information was insufficient to confirm the existence or implementation of the policy. This approach was intended to minimize the risk of overestimating the extent of policy implementation across countries.

For each country, we coded the presence of each policy as implemented or not implemented and summed these binary indicators to obtain an unweighted score ranging from 0 to 9. For pragmatic reasons, this summary measure treats all policies as equally important, given the binary or quasi-binary nature of the underlying indicators and the limited sample of 53 countries. Therefore, it should be interpreted as a crude proxy for the comprehensiveness of the national policy framework, rather than as a validated index of policy performance.

For descriptive and regression analyses, we grouped countries into three categories reflecting differences in the breadth of adoption: 1–3 policies (limited adoption across the nine domains; “low” group); 4–8 policies (substantial, yet incomplete, adoption of policies, identified as “medium” group); and 9 policies (full adoption of all measures; “high” group). These cut-points are inherently pragmatic and were not empirically optimized. To assess the sensitivity of our findings to the construction of this exposure variable, we re-estimated the models using the continuous 0–9 policy count and alternative groupings of the summary measure. The results are reported in Supplementary Table S2.

All analyses were performed using R version 4.3.0 (R Foundation for Statistical Computing, Vienna, Austria). The regression coefficients (*β*) and their corresponding confidence intervals (CIs) and *p*-values are reported. The Benjamini-Hochberg method was applied to each single battery of regression models which analyzed the same dependent variable to correct estimates accounting for the chance false discovery rate affecting results, except for the comparison by groups (see Table 1). Neverthless, uncorrected significant associations were showed in the text. Tests and estimates were calculated at 95% significance level.

**Table 1:** Adjusted multivariable regression models examining the association between policy-adoption categories and population-health outcomes.

Outcome	*N* (%)	Group	*β* (95% CI)	*p*-value
Healthy life expectancy at birth, years (year)	26	Medium vs. low	2.379 (0.053, 4.706)	0.0452
	29	High vs. low	3.349 (1.134, 5.564)	0.0039
Probability of dying from CVD, cancer, diabetes, or CRD, (year)	26	Medium vs. low	−4.948 (−10.04, 0.144)	0.0565
	29	High vs. low	−6.656 (−11.505, −1.808)	0.0082
Standardized preventable mortality, rate (year)	17	Medium vs. low	−99.493 (−313.111, 114.126)	0.3477
	17	High vs. low	−151.206 (−359.755, 57.342)	0.1484
Standardized treatable mortality, rate (year)	17	Medium vs. low	−8.469 (−115.119, 98.181)	0.8718
	17	High vs. low	−42.109 (−146.228, 62.009)	0.4139
Average length of stay, all hospitals, days (year)	27	Medium vs. low	−2.038 (−3.828, −0.247)	0.0266
	30	High vs. low	−1.866 (−3.571, −0.16)	0.0327
Surgical wound infection rate, all operations, % (year)	17	Medium vs. low	−1.620 (−3.696, 0.457)	0.1200
	17	High vs. low	−0.855 (−2.78, 1.07)	0.3671
Vaccination against influenza on average and in the poorest quintile, % (years)	16	Medium vs. low	−1.725 (−18.33, 14.879)	0.8323
	16	High vs. low	−1.358 (−17.632, 14.916)	0.8649
Under-five mortality (per 1,000 live births), 2021	29	Medium vs. low	−5.229 (−9.73, −0.727)	0.0238
	32	High vs. low	−7.521 (−11.767, −3.274)	0.0009
Maternal mortality (per 100,000 live births), 2020	26	Medium vs. low	1.557 (−12.015, 15.129)	0.8183
	29	High vs. low	0.536 (−12.387, 13.459)	0.9338

Beta coefficients represent the difference in each outcome variable for countries in the medium category (adoption of 4–8 policies) or the high category (full adoption of all nine policies), compared with the low category (adoption of 1–3 policies), which served as the reference group. Models were adjusted for the number of medical doctors per 10,000 population per year, nursing personnel per 10,000 population per year, and public health expenditure as a proportion of total public expenditure per year. CI, confidence interval.

## Results

### Regression analysis

According to the analysis of individual policies (see Table 2), the implementation of a national quality of care plan was found to be significantly associated with an increase in healthy life expectancy of 1.68 years (*p*unadjusted = 0.033, *p* = 0.144). The plan was also linked to a 3.33 percentage point reduction in the probability of premature death from cardiovascular disease, cancer, diabetes, and chronic respiratory diseases (*p*unadjusted = 0.049, *p* = 0.144). Furthermore, the analysis revealed a reduction in under-five mortality of 4.06 per 1,000 live births (*p*unadjusted = 0.023, *p* = 0.144).

**Table 2:** Adjusted multivariable regression models for each national plan and selected population-health outcomes.

Trait	*N*	Predictors																	
		National QoC plan		National patient safety plan		National antimicrobial resistance plan		Health misinformation prevention plan		Accreditation systems for hospitals		Patient public representation in national health governance		National electronic health record system		Quality and safety in telehealth guidelines		National list of approved priority essential medical devices	
		Adjusted estimates		Adjusted estimates		Adjusted estimates		Adjusted estimates		Adjusted estimates		Adjusted estimates		Adjusted estimates		Adjusted estimates		Adjusted estimates	
		*β* (95% CI)	*p*-value	*β* (95% CI)	*p*-value	*β* (95% CI)	*p*-value	*β* (95% CI)	*p*-value	*β* (95% CI)	*p*-value	*β* (95% CI)	*p*-value	*β* (95% CI)	*p*-value	*β* (95% CI)	*p*-value	*β* (95% CI)	*p*-value
Dependent variables																			
Healthy life expectancy at birth, years (year)	50	1.68 (0.15 to 3.22)	0.144	1.08 (−0.62 to 2.78)	0.347	1.16 (−3.74 to 6.05)	0.763	1.33 (0.06 to 2.60)	0.180	1.00 (−0.44 to 2.45)	0.509	0.84 (−0.65 to 2.33)	0.411	1.16 (−3.74 to 6.05)	0.636	0.77 (−0.62 to 2.16)	0.731	2.17 (−0.58 to 4.92)	0.537
Probability of dying from CVD, cancer, diabetes, or CRD, (year)	50	−3.33 (−6.66 to −0.01)	0.144	−2.51 (−6.15 to 1.13)	0.347	−2.66 (−13.17 to 7.85)	0.763	−2.50 (−5.26 to 0.25)	0.189	−2.17 (−5.27 to 0.94)	0.509	−1.95 (−5.15 to 1.24)	0.411	−2.65 (−13.17 to 7.85)	0.613	−1.37 (−4.37 to 1.63)	0.731	−4.03 (−9.98 to −1.92)	0.537
Standardized preventable mortality, rate (year)	33	−53.51 (−144.95 to 2.12)	0.434	−72.80 (−172.83 to 27.23)	0.347	n.e.		−51.95 (−122.27 to 18.36)	0.254	−34.80 (−118.01 to 48.41)	0.663	−63.40 (−142.91 to 16.11)	0.411	n.e.		−31.87 (−109.18 to 45.44)	0.731	−45.85 (−268.38 to 176.67)	0.890
Standardized treatable mortality, rate (year)	33	−22.46 (−68.48 to 23.57)	0.489	−36.13 (−86.13 to 13.87)	0.347	n.e.		−30.23 (−64.84 to 4.38)	0.189	−24.78 (−65.79 to 16.23)	0.509	−33.82 (−73.28 to 5.64)	0.411	n.e.		−22.56 (−60.68 to 15.56)	0.731	−21.75 (−132.96 to 89.46)	0.890
Average length of stay, all hospitals, days (year)	51	−1.19 (−2.45 to 0.07)	0.144	−1.11 (−2.40 to 0.18)	0.347	−3.25 (−5.99 to −0.50)	0.132	−0.45 (−1.53 to 0.62)	0.602	−0.18 (−1.37 to 1.02)	0.800	−0.68 (−1.84 to 0.53)	0.411	−3.25 (−5.96 to −0.50)	0.022	0.04 (−1.08 to 1.17)	0.940	0.74 (−2.07 to 3.55)	0.644
Surgical wound infection rate, all operations, % (year)	28	−0.39 (−2.05 to 1.28)	0.738	−0.73 (−2.49 to 1.03)	0.451	2.77 (−3.10 to 8.64)	0.763	0.08 (−1.35 to 1.52)	0.904	−0.19 (−1.75 to 1.36)	0.800	−0.19 (−1.75 to 1.36)	0.800	2.77 (−3.10 to 8.64)	0.339	−0.29 (−1.72 to 1.14)	0.874	0.07 (−2.77 to 2.90)	0.960
Vaccination against influenza on average and in the poorest quintile, % (years)	31	1.51 (−5.36 to 8.37)	0.738	3.65 (−4.00 to 11.31)	0.431	n.e.		1.01 (−4.57 to 6.59)	0.904	−2.33 (−8.47 to 3.81)	0.663	2.89 (−3.13 to 8.91)	0.428	n.e.		0.77 (−5.34 to 6.89)	0.896	−7.01 (−23.34 to 9.32	0.695
Under-five mortality (per 1,000 live births), 2021	53	−4.06 (−7.53 to −0.59)	0.144	−2.23 (−5.92 to 1.47)	0.347	−0.48 (−7.47 to 6.51)	0.890	−3.48 (−6.48 to −0.47)	0.180	−3.42 (−6.67 to −0.16)	0.360	−2.27 (−5.64 to 1.11)	0.411	−2.24 (−10.92 to 6.45)	0.607	−3.00 (−6.16 to 0.16)	0.558	−8.56 (−14.10 to −3.03)	0.027
Maternal mortality (per 100,000 live births), 2020	50	0.21 (−8.30 to 8.72)	0.961	1.65 (−7.46 to 10.76)	0.717	8.16 (−17.59 to 33.91)	0.763	−0.43 (−7.44 to 6.59)	0.904	1.79 (−5.97 to 9.56)	0.800	2.21 (−5.73 to 10.15)	0.650	8.16 (−17.59 to 33.91)	0.527	−1.69 (−9.10 to 5.71)	0.874	−0.73 (−15.64 to 14.17)	0.960

Each beta coefficient represents the change in the dependent variable (listed in the “Trait” column) associated with a one-unit increase in the respective independent variable (e.g., national QoC plan, national patient safety plan). Models were adjusted for the number of medical doctors per 10,000 population per year, nursing personnel per 10,000 population per year, and public health expenditure as a proportion of total public expenditure per year. The Benjamini–Hochberg correction was applied to account for multiple testing. CI, confidence interval; n.e., not evaluated.

Using a national antimicrobial resistance plan was significantly associated with reducing the average hospital length of stay by 3.25 days (*p*unadjusted = 0.022, *p* = 0.132). Meanwhile, the health misinformation prevention plan was linked to an increase in healthy life expectancy of 1.33 years (*p*unadjusted = 0.040, *p* = 0.180) and a reduction in under-five mortality of 3.48 per 1,000 live births (*p*unadjusted = 0.024, *p* = 0.180). Hospital accreditation systems were associated with a 3.42 per 1,000 reduction in under-five mortality (*p*unadjusted = 0.040, *p* = 0.360). Implementing a national electronic health record system was significantly associated with reducing the average hospital stay by 3.25 days (*p*unadjusted = 0.022, *p* = 0.132). Furthermore, the presence of a national list of approved essential medical devices was strongly associated with an 8.56 per 1,000 live births reduction in under-five mortality, estimate which kept the statistical significance after correcting for multiple testing (*p*unadjusted = 0.003, and *p* = 0.020, respectively).

Table 3 summarizes the associations for the policy count across outcomes. Implementing multiple national policies was associated with an increase in healthy life expectancy of 0.29 years (*p*unadjusted = 0.043) and a reduction in under-five mortality of 0.72 per 1,000 live births (*p* = 0.018), estimates which lost their significance when accounting for multiple testing. While not statistically significant, several policies demonstrated promising trends, including reductions in preventable and treatable mortality, shorter hospital stays, lower rates of surgical wound infection, and modest improvements in vaccination coverage.

**Table 3:** Adjusted multivariable regression models examining the association between the number of national action plans (independent variable) and individual population-health indicators (listed in the “Trait” column).

Trait	*N*	National action plans and policies for quality of care and patient safety (per increase of one plan of policy)	
Dependent variables		Adjusted estimates	
		*β* (95% CI)	*p*-value
Healthy life expectancy at birth, years (year)	50	0.29 (0.01 to 0.57)	0.167
Probability of dying from CVD, cancer, diabetes, or CRD, (year)	50	−0.59 (−1.20 to 0.02)	0.167
Standardized preventable mortality, rate (year)	33	−13.46 (−30.43 to 3.51)	0.207
Standardized treatable mortality, rate (year)	33	−7.57 (−15.96 to 0.79)	0.167
Average length of stay, all hospitals, days (year)	51	−0.15 (−0.37 to 0.07)	0.270
Surgical wound infection rate, all operations, % (year)	28	−0.04 (−0.32 to 0.25)	0.830
Vaccination against influenza on average and in the poorest quintile, % (years)	31	0.24 (−1.07 to 1.57)	0.830
Under-five mortality (per 1,000 live births), 2021	53	−0.72 (−1.31 to −0.13)	0.162
Maternal mortality (per 100,000 live births), 2020	50	0.17 (−1.39 to 1.73)	0.830

Models were adjusted for the number of medical doctors per 10,000 population per year, nursing personnel per 10,000 population per year, and public health expenditure as a proportion of total public expenditure per year. The Benjamini–Hochberg correction was applied to account for multiple testing. CI, confidence interval.

Table 1 presents the results of multiple linear regression models that investigated the association between public health indicators and the level of policy implementation, based on the total number of policies adopted. These models used the same covariate set as described in the Methods. Including these covariates enabled us to isolate the specific effect of policy implementation levels on health outcomes while accounting for differences in healthcare resources across countries. The results show that healthy life expectancy is significantly higher in countries with medium and high levels of policy implementation than in those with low levels, with estimated increases of approximately 2.4 years (*p* = 0.045) and 3.3 years (*p* = 0.004), respectively. The probability of dying from major chronic diseases is significantly lower in the ‘high’ group than in the ‘low’ group (−6.7%, *p* = 0.008), and a similar, albeit non-significant, trend was observed in the “medium” group (*p* = 0.057). No significant associations were found for preventable and treatable mortality rates, although negative coefficients suggest a possible downward trend. The average hospital stay was significantly shorter in the “medium” group (by 2.0 days, *p* = 0.027) and the “high” group (by 1.9 days, *p* = 0.033) than in the “low” group. Other indicators, such as surgical wound infection rates and influenza vaccination coverage, showed no significant differences across implementation levels. However, under-five mortality was significantly lower in the “medium” group (5.2 per 1,000 live births, *p* = 0.024) and the “high” group (7.5 per 1,000 live births, *p* < 0.001). Maternal mortality did not differ significantly across groups.

Figure 1 illustrates the distribution of health policies adopted by WHO European Region Member States. Countries are represented by dots, which are positioned according to the exact number of policies implemented. The color of each dot indicates a predefined adoption category: low (1–3 policies), medium (4–8 policies) or high (9 policies). The majority of countries (24, or 45.3%) fall within the high adoption category, with fewer countries in the low (8, or 15.1%) or medium (21, or 39.6%) adoption groups.

**Figure 1:**
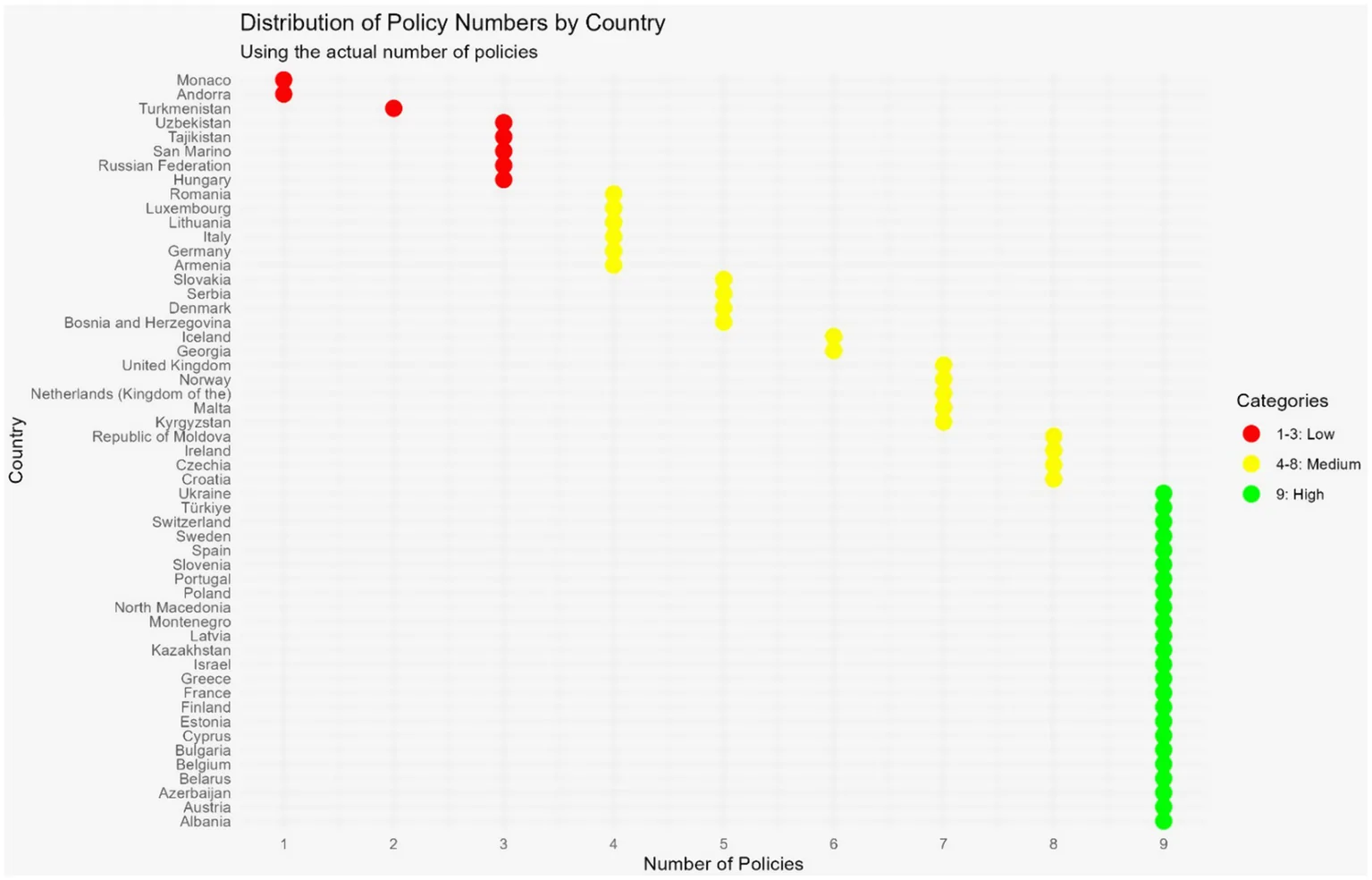
Distribution of the number of adopted health policies across countries.

Notably, a group of countries implemented all nine policies, representing the high adoption category. This distribution highlights the substantial variation in the comprehensiveness of national health policy implementation between countries. Figure 2 shows the grouping of WHO European Region Member States according to their level of policy adoption; countries are arranged alphabetically. This organization provides a clearer view of which countries fall within each adoption category, emphasizing both the variability within groups and the distribution of countries across levels of implementation.

**Figure 2:**
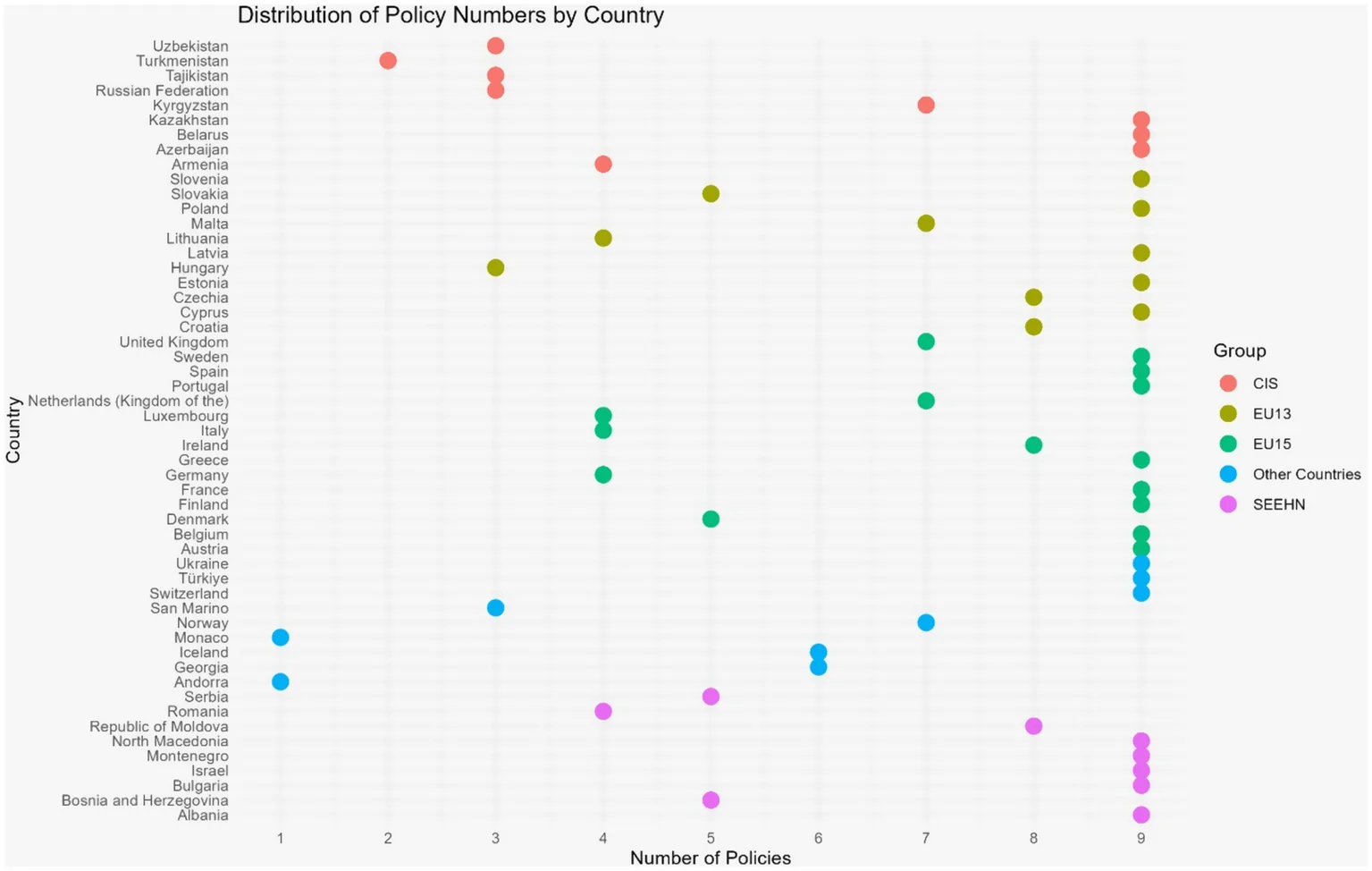
Countries grouped by level of adoption of health policies (countries group graph).

These findings suggest that higher levels of health policy addressing quality of care and patient safety implementation are associated with more favorable levels of key health indicators, particularly healthy life expectancy, mortality from chronic diseases, under-five mortality and average hospital stay, although many associations for other outcomes were small in magnitude and not statistically significant.

In sensitivity analyses using alternative policy-group cut-offs and log-transformed outcomes, the direction and approximate magnitude of the associations for healthy life expectancy and under-five mortality were similar to those observed in the main models. On this basis, we focus on the continuous 0–9 policy count in the main text, while treating all model specifications as exploratory and subject to residual confounding.

## Discussion

The main contribution of this study is to provide a comparable, region-wide snapshot of the adoption of national quality-of-care and patient-safety policies and to explore, in a transparent and hypothesis-generating manner, how the breadth of policy adoption aligns with selected population health outcomes. Overall, countries in the WHO European Region reporting more comprehensive policy adoption tended to report more favorable levels of several health indicators. Analysis of individual policies showed that the presence of a national quality-of-care plan was associated with higher healthy life expectancy (+1.68 years) and lower premature mortality from major chronic diseases (−3.33 percentage points) and under-five mortality (−4.06 per 1,000 live births). National antimicrobial-resistance plans and national electronic health-record systems were associated with shorter average hospital stays. Hospital accreditation systems and national lists of approved essential medical devices were also associated with lower under-five mortality, while health misinformation prevention plans were associated with higher healthy life expectancy and lower under-five mortality. These findings describe country-level associations and should not be interpreted as evidence that the implementation of any individual policy caused the observed differences in outcomes.

The observed patterns may be compatible with differences in national policy environments, including care coordination, infection prevention, data and information systems, regulatory arrangements, access to essential technologies, and health communication. However, the present ecological cross-sectional analysis cannot assess the mechanisms underlying these patterns, establish the temporal sequence between policy adoption and observed outcomes, or distinguish the contribution of individual policies from that of wider health-system, socioeconomic, institutional, and governance characteristics. The findings should therefore be interpreted as descriptive country-level patterns that generate hypotheses for further investigation rather than as estimates of the independent effects of particular policies.

The cumulative analyses showed a similar pattern: countries reporting a greater number of implemented policies tended to have more favorable values for several population health indicators, including healthy life expectancy and under-five mortality. The aggregate policy count was developed as a pragmatic proxy for the breadth of national policy adoption across the nine domains covered by the survey. It was not designed as a validated measure of policy quality, implementation intensity, duration, enforcement, or effectiveness, and each policy domain was assigned equal weight. Countries reporting implementation of all nine policies generally had more favorable outcomes than countries reporting fewer policies, whereas countries with limited policy adoption tended to have less favorable outcomes. These patterns may reflect more comprehensive policy environments, but they may also reflect wider differences between countries that were not fully captured in the analysis.

For several other indicators—including preventable and treatable mortality, surgical wound infection rates, maternal mortality, and influenza vaccination coverage—the estimated country-level associations did not reach statistical significance. Although some estimates were similar in direction to those observed for healthy life expectancy, under-five mortality, or hospital length of stay, they should be interpreted with particular caution. Non-significant findings may reflect limited statistical power, random variation, residual or unmeasured confounding, differences in outcome measurement and data availability across countries, or genuinely weak or absent associations. They should not be interpreted as evidence of beneficial policy effects, but rather as exploratory patterns requiring further investigation.

The direction and approximate magnitude of several associations remained broadly consistent after adjustment for physician density, nursing personnel density, and public expenditure on health. This adjustment accounted for selected differences in health-system resources across countries; however, persistence of the associations after adjustment does not demonstrate that they are independent of broader contextual factors. The adjusted estimates should therefore be understood as descriptive ecological associations rather than isolated or causal effects of policy implementation.

Nevertheless, the observed country-level patterns may provide a useful basis for policy dialog, peer learning, and the identification of questions for future research. Countries at different stages of policy adoption may draw on one another’s experience in developing national quality-of-care and patient-safety frameworks, while recognizing that this analysis cannot establish whether particular policies, or a greater number of policies, account for differences in outcomes between countries.

The statistical analysis has several limitations that should be considered when interpreting the findings. First, the ecological cross-sectional design can identify associations between country-level policy environments and population-level health indicators, but it cannot establish temporal relationships or causal effects. Because exposures, covariates, and outcomes were measured at the aggregate country level, the results cannot be used to assess risks, effects, or mechanisms within specific population subgroups or at the level of individual patients, healthcare professionals, hospitals, or other healthcare facilities. Accordingly, associations observed between countries should not be assumed to represent equivalent associations at individual or service-delivery level, and the potential for ecological fallacy should be recognized.

Second, the relatively small sample of 53 countries, together with the number of policy measures, outcomes, and covariates considered, limited statistical power, particularly for detecting modest associations. The available sample also constrained the complexity of the regression models and required a parsimonious adjustment strategy. As a result, confidence intervals for some estimates were wide, and several associations that were statistically significant before correction were attenuated after applying procedures for multiple testing. The absence of statistically significant associations for some outcomes should not necessarily be interpreted as evidence that no relationship exists; equally, observed associations should not be considered evidence of policy effects without confirmation in studies using repeated observations and more detailed implementation data.

Third, multiple regression models were estimated across several policy measures and population health outcomes. Although the Benjamini–Hochberg procedure was applied within each set of analyses examining the same outcome, the large number of comparisons means that some nominally statistically significant findings may have occurred by chance. This issue is particularly relevant for the analyses of individual policies, for which several associations did not remain statistically significant after adjustment for multiple testing. The results should therefore be considered exploratory and hypothesis-generating rather than confirmatory, and emphasis should be placed on the overall pattern and consistency of findings rather than on isolated estimates or nominal *p*-values.

Finally, although the regression models adjusted for physician density, nursing personnel density, and public expenditure on health, these variables capture only a limited part of the broader context in which national policies are adopted and implemented. Differences between countries in health-system development, national income, education, social protection, governance, institutional capacity, political commitment, policy implementation quality and duration, and the completeness and comparability of health data may influence both policy adoption and the outcomes examined. Residual and unmeasured confounding therefore remain likely, and reverse causation cannot be excluded: countries with stronger health systems or more favorable health outcomes may also be better placed to adopt, implement, monitor, and report comprehensive quality-of-care and patient-safety policies.

Taken together, these limitations mean that the estimated coefficients should be interpreted as descriptive ecological associations between national policy environments and population health indicators, rather than as definitive estimates of the independent or causal effects of individual policies. The findings provide a basis for hypothesis generation and for future research using longitudinal, panel-data, quasi-experimental, and more granular individual-, facility-, or health-system-level designs to examine implementation processes, temporal relationships, and potential causal pathways more rigorously.

## Policy implications and leadership perspective

Our study suggests that countries with more comprehensive national quality-of-care and patient-safety policies tend to report more favorable population health indicators. However, these country-level ecological associations do not establish that specific policies caused the observed differences or that they apply at individual, provider, hospital, or facility level. The patterns are consistent with WHO guidance and may reflect leadership and governance, but also wider differences in health-system development, resources, institutional capacity, socioeconomic conditions, governance, and data quality. Residual confounding and reverse causation cannot be excluded.

These exploratory, hypothesis-generating findings may inform policy dialog and peer learning, but require confirmation through longitudinal, quasi-experimental, and more granular research.

## Data Availability

The raw data supporting the conclusions of this article will be made available by the authors, without undue reservation.
