# Supplementary material for: Use of quality-of-care and patient-safety policies in the WHO European region: an ecological cross-sectional exploratory analysis of policy adoption and health outcomes
Source: Front Public Health. 2026 Oct 12;14:1904393. doi: 10.3389/fpubh.2026.1904393 (PMC13696396; doi:10.3389/fpubh.2026.1904393)
Supplement: Supplementary file 1 [file Data_Sheet_1.docx]

**Supplementary Section:**

To evaluate the robustness of the findings with respect to outcome distributions and model assumptions, we examined the distributional properties of all dependent variables. Those showing substantial positive skewness (skewness >1) were log-transformed prior to analysis. This included the average length of stay in all hospitals, the surgical wound infection rate, the under-five mortality rate and the maternal mortality rate.

The primary regression models were re-estimated for each log-transformed outcome using the same specification as in the main analyses. Additionally, all regression models were re-estimated using heteroskedasticity-robust standard errors, in order to account for any potential violations of the constant variance assumption.

Supplementary **Table S1** reports the regression coefficients, 95% confidence intervals, and p-values from the models estimated with log-transformed outcomes and heteroskedasticity-robust standard errors. To assess the robustness of the findings across specifications, we compared the direction, magnitude, and statistical significance of the estimated associations with those from the primary analyses.

**Table S1**. Adjusted multivariate regression models were conducted for each national plan with a skewed distribution and for selected population health outcomes. Each beta represents the change in the dependent variable (e.g., national QoC plan, national patient safety plan, or national antimicrobial resistance plan) associated with a one-unit increase in the log-transformed independent variable listed in the “Trait” column. Models were adjusted for medical doctors per 10,000 population per year, nursing personnel per 10,000 population per year, and public spending on health as a proportion of total public spending per year. The Benjamini–Hochberg correction for multiple testing was applied. Abbreviations: CI, confidence interval; n.e., not evaluated.

| **Trait** | **National QoC plan** | | **National patient safety plan** | | **National antimicrobial resistance plan** | | **Health misinformation prevention plan** | | **Accreditation systems for hospitals** | |
| --- | --- | --- | --- | --- | --- | --- | --- | --- | --- | --- |
|  | **Adjusted estimates** | | **Adjusted estimates** | | **Adjusted estimates** | |  | |  | |
|  | **Beta (95% CI)** | **p-value** | **Beta (95% CI)** | **p-value** | **Beta (95% CI** | **p-value** | **Beta (95% CI** | **p-value** | **Beta (95% CI** | **p-value** |
| **Log-Average length of stay, all hospitals, days (year)** | -0.13 (-0.26 to 0.01) | 0.234 | -0.11 (-0.25 to 0.03) | 0.448 | -0.23 (-0.54 to 0.08) | 0.552 | -0.05 (-0.16 to 0.07) | 0.564 | -0.01 (-0.14 to 0.12) | 0.988 |
| **Log-Surgical wound infection rate, all operations, % (year)** | 0.05 (-0.39 to 0.49) | 0.816 | -0.05 (-0.52 to 0.43) | 0.841 | 0.47 (-1.11 to 2.05) | 0.792 | 0.15 (-0.23 to 0.52) | 0.564 | 0.08 (-0.33 to 0.49) | 0.988 |
| **Log-Under-five mortality (per 1000 live births), 2021** | -0.21 (-0.48 to 0.06) | 0.234 | -0.03 (-0.32 to 0.25) | 0.841 | -0.05 (-0.58 to 0.47) | 0.841 | -0.29 (-0.51 to -0.06) | 0.052 | -0.20 (-0.45 to 0.05) | 0.488 |
| **Log-Maternal mortality (per 100 000 live births), 2020** | -0.15 (-0.62 to 0.33) | 0.713 | -0.15 (-0.66 to 0.36) | 0.841 | 0.39 (-1.06 to 1.83) | 0.792 | -0.10 (-0.49 to 0.29) | 0.614 | 0.00 (-0.43 to 0.44) | 0.988 |

**Table S1. continued.**

| **Trait** | **Patient public representation in national health governance** | | **National electronic health record system** | | **Quality and safety in telehealth guidelines** | | **National list of approved priority essential medical devices** | |
| --- | --- | --- | --- | --- | --- | --- | --- | --- |
|  | **Adjusted estimates** | | **Adjusted estimates** | | **Adjusted estimates** | |  | |
|  | **Beta (95% CI)** | **p-value** | **Beta (95% CI)** | **p-value** | **Beta (95% CI** | **p-value** | **Beta (95% CI** | **p-value** |
| **Log-Average length of stay, all hospitals, days (year)** | -0.06 (-0.19 to 0.07) | 0.790 | -0.23 (-0.54 to 0.08) | 0.552 | 0.01 (-0.11 to 0.13) | 0.832 | 0.09 (-0.22 to 0.39) | 0.864 |
| **Log-Surgical wound infection rate, all operations, % (year)** | 0.08 (-0.33 to 0.49) | 0.790 | 0.47 (-1.11 to 2.05) | 0.594 | 0.06 (-0.32 to 0.44) | 0.832 | 0.06 (-0.69 to 0.82) | 0.864 |
| **Log-Under-five mortality (per 1000 live births), 2021** | -0.08 (-0.34 to 0.17) | 0.790 | -0.31 (-0.96 to 0.34) | 0.594 | -0.17 (-0.41 to 0.07) | 0.672 | -0.45 (-0.89 to -0.02) | 0.168 |
| **Log-Maternal mortality (per 100 000 live births), 2020** | -0.06 (-0.51 to 0.39) | 0.790 | 0.39 (-1.06 to 1.83) | 0.594 | -0.16 (-0.57 to 0.26) | 0.832 | -0.07 (-0.91 to 0.77) | 0.864 |

**To evaluate the robustness of the findings with regard to the operationalisation of policy exposure, we conducted sensitivity analyses using an alternative categorisation of the 0–9 policy count, alongside the continuous specification underlying the primary 'per additional policy' estimates.**

**However, the distribution of policy counts in our sample imposed important constraints on the choice of alternative groupings. Of the 53 countries included, 24 (45.3%) had a policy count of exactly nine, reflecting the full adoption of all nine evaluated policies. This marked right-skewness made a tertile-based categorisation methodologically infeasible, as countries with a policy count of nine were automatically assigned to multiple tertile groups, preventing meaningful comparisons between groups.**

**Therefore, one alternative categorical grouping was implemented alongside the original categorisation (1–3 / 4–8 / 9): a three-level grouping with revised cut-points (1–3 / 4–7 / 8–9). This yielded groups of n = 8, 17 and 28 countries, respectively. All models were estimated using the same set of covariates as in the primary analyses.**

**Supplementary Table S2 reports the regression coefficients, 95% confidence intervals and p-values for both exposure specifications. The direction and magnitude of the associations were consistent across the two groupings, supporting the robustness of the categories reported in the paper (1–3 / 4–8 / 9).**

**Table S2.** Adjusted multivariate regression models between policies categories and selected population health outcomes. Betas refer to the variation of the value in each single outcome variable in the “Medium” or the “High” groups, compared to the “Low” one the reference category within the two different groupings. Models were adjusted for terms of: *Medical doctors per 10000 population year; Nursing personnel per 10000 population year; Public spending on health as of total public spending year.* Abbreviation: CI, confidence interval.

| **Outcome** | **Group** | **Comparison** | |
| --- | --- | --- | --- |
|  |  | **Original groupings** (**Table 3** of the main**)**  **(1–3 / 4–8 / 9)** | **Alternative groupings**  **(1–3 / 4–7 / 8–9)** |
|  |  | **β (95% CI)**  **p-value** | **β (95% CI)**  **p-value** |
| Healthy life expectancy at birth, years | *Medium vs Low* | 2.379 (0.053–4.706) p=0.045* | 2.789 (0.390–5.188) p=0.024* |
|  | *High vs Low* | 3.349 (1.134–5.564) p=0.004* | 3.104 (0.850–5.359) p=0.008* |
| Probability of dying from CVD, cancer, diabetes, or CRD | *Medium vs Low* | −4.948 (−10.040–0.144) p=0.057 | −5.676 (−10.887– −0.465) p=0.033* |
|  | *High vs Low* | −6.656 (−11.505– −1.808) p=0.008* | −6.223 (−11.120– −1.326) p=0.014* |
| Standardized preventable mortality, rate | *Medium vs Low* | −99.493 (−313.111–114.126) p=0.348 | −118.124 (−337.743–101.495) p=0.280 |
|  | *High vs Low* | −151.206 (−359.755–57.342) p=0.148 | −142.921 (−357.384–71.542) p=0.183 |
| Standardized treatable mortality, rate | *Medium vs Low* | −8.469 (−115.119–98.181) p=0.872 | −15.470 (−125.443–94.504) p=0.775 |
|  | *High vs Low* | −42.109 (−146.228–62.009) p=0.414 | −38.904 (−146.296–68.487) p=0.464 |
| Average length of stay, all hospitals, days | *Medium vs Low* | −2.038 (−3.828– −0.247) p=0.027* | −2.195 (−3.998– −0.391) p=0.018* |
|  | *High vs Low* | −1.866 (−3.571– −0.160) p=0.033* | −1.796 (−3.482– −0.109) p=0.037* |
| Surgical wound infection rate, all operations, % | *Medium vs Low* | −1.620 (−3.696–0.457) p=0.120 | −1.439 (−3.586–0.709) p=0.179 |
|  | *High vs Low* | −0.855 (−2.780–1.070) p=0.367 | −0.997 (−2.932–0.938) p=0.297 |
| Vaccination against influenza, average and poorest quintile, % | *Medium vs Low* | −1.725 (−18.330–14.879) p=0.832 | −1.743 (−18.337–14.851) p=0.831 |
|  | *High vs Low* | −1.358 (−17.632–14.916) p=0.865 | −1.343 (−17.624–14.939) p=0.867 |
| Under-five mortality (per 1,000 live births), 2021 | *Medium vs Low* | −5.229 (−9.730– −0.727) p=0.024* | −5.875 (−10.531– −1.218) p=0.015* |
|  | *High vs Low* | −7.521 (−11.767– −3.274) p=0.001* | −7.010 (−11.283– −2.738) p=0.002* |
| Maternal mortality (per 100,000 live births), 2020 | *Medium vs Low* | 1.557 (−12.015–15.129) p=0.818 | 2.151 (−11.518–15.820) p=0.753 |
|  | *High vs Low* | 0.536 (−12.387–13.459) p=0.934 | 0.318 (−12.525–13.161)  P=0.96 |
